# Thiopurines Activate an Antiviral Unfolded Protein Response That Blocks Influenza A Virus Glycoprotein Accumulation

**DOI:** 10.1128/JVI.00453-21

**Published:** 2021-05-10

**Authors:** Patrick D. Slaine, Mariel Kleer, Brett A. Duguay, Eric S. Pringle, Eileigh Kadijk, Shan Ying, Aruna Balgi, Michel Roberge, Craig McCormick, Denys A. Khaperskyy

**Affiliations:** a Department of Microbiology & Immunology, Dalhousie University, Halifax, Nova Scotia, Canada; b Department of Microbiology, Immunology and Infectious Diseases, University of Calgary, Calgary, Alberta, Canada; c Department of Biochemistry and Molecular Biology, University of British Columbia, Vancouver, British Columbia, Canada; St. Jude Children’s Research Hospital

**Keywords:** virus, influenza, thiopurine, 6-thioguanine, 6-thioguanosine, IRE1, XBP1, PERK, hemagglutinin, neuraminidase, glycosylation, host-targeted antiviral

## Abstract

Influenza A viruses (IAVs) utilize host shutoff mechanisms to limit antiviral gene expression and redirect translation machinery to the synthesis of viral proteins. Previously, we showed that IAV replication is sensitive to protein synthesis inhibitors that block translation initiation and induce the formation of cytoplasmic condensates of untranslated messenger ribonucleoprotein complexes called stress granules (SGs). In this study, using an image-based high-content screen, we identified two thiopurines, 6-thioguanine (6-TG) and 6-thioguanosine (6-TGo), that triggered SG formation in IAV-infected cells and blocked IAV replication in a dose-dependent manner without eliciting SG formation in uninfected cells. 6-TG and 6-TGo selectively disrupted the synthesis and maturation of the IAV glycoproteins hemagglutinin (HA) and neuraminidase (NA) without affecting the levels of the viral RNAs that encode them. By contrast, these thiopurines had a minimal effect on other IAV proteins or global host protein synthesis. Disruption of IAV glycoprotein accumulation by 6-TG and 6-TGo correlated with the activation of the unfolded protein response (UPR) sensors activating transcription factor 6 (ATF6), inositol-requiring enzyme 1 (IRE1), and double-stranded RNA-activated protein kinase (PKR)-like endoplasmic reticulum (ER) kinase (PERK), leading to downstream UPR gene expression. Treatment of infected cells with the chemical chaperone 4-phenylbutyric acid diminished thiopurine-induced UPR activation and partially restored the processing and accumulation of HA and NA. By contrast, chemical inhibition of the integrated stress response downstream of PERK restored the accumulation of NA monomers but did not restore the processing of viral glycoproteins. Genetic deletion of PERK enhanced the antiviral effect of 6-TG without causing overt cytotoxicity, suggesting that while UPR activation correlates with diminished viral glycoprotein accumulation, PERK could limit the antiviral effects of drug-induced ER stress. Taken together, these data indicate that 6-TG and 6-TGo are effective host-targeted antivirals that trigger the UPR and selectively disrupt the accumulation of viral glycoproteins.

**IMPORTANCE** Secreted and transmembrane proteins are synthesized in the endoplasmic reticulum (ER), where they are folded and modified prior to transport. Many viruses rely on the ER for the synthesis and processing of viral glycoproteins that will ultimately be incorporated into viral envelopes. Viral burden on the ER can trigger the unfolded protein response (UPR). Much remains to be learned about how viruses coopt the UPR to ensure efficient synthesis of viral glycoproteins. Here, we show that two FDA-approved thiopurine drugs, 6-TG and 6-TGo, induce the UPR, which represents a previously unrecognized effect of these drugs on cell physiology. This thiopurine-mediated UPR activation blocks influenza virus replication by impeding viral glycoprotein accumulation. Our findings suggest that 6-TG and 6-TGo may have broad antiviral effects against enveloped viruses that require precise tuning of the UPR to support viral glycoprotein synthesis.

## Abstract (precis)

Secreted and transmembrane proteins are synthesized in the endoplasmic reticulum (ER), where they are folded and modified prior to transport. Many viruses rely on the ER for the synthesis and processing of viral glycoproteins that will ultimately be incorporated into viral envelopes.

## INTRODUCTION

Enveloped viruses encode integral membrane proteins that are synthesized and posttranslationally modified in the endoplasmic reticulum (ER) prior to transport to sites of virion assembly. When ER protein folding capacity is exceeded, the accumulation of unfolded proteins in the ER activates the unfolded protein response (UPR), whereby activating transcription factor 6 (ATF6), inositol-requiring enzyme 1 (IRE1), and double-stranded RNA-activated protein kinase (PKR)-like endoplasmic reticulum kinase (PERK) sense ER stress and trigger a downstream transcriptional response (1). UPR gene expression causes the accumulation of proteins that attempt to restore ER proteostasis by expanding ER folding capacity and catabolic activities like ER-associated degradation (ERAD) (2). ERAD ensures that improperly folded integral membrane proteins are ubiquitinated and retrotranslocated out of the ER for degradation by the 26S proteasome. There is accumulating evidence that bursts of viral glycoprotein synthesis can burden ER protein folding machinery and that enveloped viruses subvert the UPR to promote efficient viral replication (3, 4).

Influenza A viruses (IAVs) encode three integral membrane proteins: hemagglutinin (HA), neuraminidase (NA), and matrix protein 2 (M2). HA adopts a type I transmembrane topology in the ER, followed by the addition of N-linked glycans, disulfide bond formation, and trimerization prior to transport to the Golgi apparatus and further processing by proteases and glycosyltransferases (5–11); NA adopts a type II transmembrane topology in the ER, is similarly processed by glycosyltransferases and protein disulfide isomerases, and assembles into tetramers prior to traversing the secretory pathway to the cell surface (12, 13). The small M2 protein also forms disulfide-linked tetramers in the ER, which is a prerequisite for its viroporin activity (14–16). Previous studies provided evidence for limited activation of the UPR in IAV-infected cells; IRE1 is activated, while PERK and ATF6 are not (17). Selective chemical inhibition of IRE1 activity inhibited IAV replication, suggesting that IRE1 has proviral effects. Little is known about how NA and M2 proteins affect the UPR, but HA was shown to be sufficient to activate the UPR and to be subsequently degraded by ERAD (18). IAV has also been shown to be susceptible to subcytotoxic doses of thapsigargin (Tg), a broad activator of the UPR that inhibits sarco/endoplasmic reticulum calcium ATPase (SERCA) and depletes ER calcium stores (19). Together, these reports suggest that efficient IAV replication may rely upon fine-tuning of the UPR.

Viral mRNAs are decoded by the host protein synthesis machinery, which makes them subject to stress-induced regulation of protein synthesis. In addition to ER stress-mediated activation of PERK, three other sentinel kinases (PKR, heme-regulated translation inhibitor [HRI], and general control nonderepressible 2 [GCN2]) respond to diverse stresses by phosphorylating eukaryotic translation initiation factor 2 alpha (eIF2α) and enforcing a translation initiation checkpoint (20). The assembly of the ternary complex, comprised of eIF2, GTP, and Met-tRNAi^met^, is essential for the incorporation of Met-tRNAi^met^ into the 40S ribosomal subunit during translation initiation. Phosphorylated eIF2α binds to the eIF2B guanine nucleotide exchange factor and prevents it from exchanging GTP for GDP and recharging the ternary complex. This leads to global inhibition of translation initiation while favoring the synthesis of proteins encoded by mRNAs that contain short upstream open reading frames (uORFs) (e.g., activating transcription factor 4 [ATF4] that drives integrated stress response [ISR] gene expression, CCAAT enhancer binding protein [C/EBP]-homologous protein [CHOP], and growth arrest and DNA damage 34 [GADD34]). Inhibition of global protein synthesis causes the accumulation of 43S preinitiation complexes and associated mRNAs that recruit aggregation-prone RNA binding proteins that mediate the condensation of cytoplasmic stress granules (SGs). Thus, ISR activation threatens efficient viral protein synthesis and causes the formation of SGs (21). Many viruses have acquired the means to prevent translation arrest and SG formation and thereby limit the negative impact of the ISR on viral protein synthesis (20).

We previously demonstrated that IAV suppresses SG formation in infected cells at later times postinfection due to the accumulation of three viral proteins with SG-suppressing activity: nonstructural protein 1 (NS1), nucleoprotein (NP), and polymerase acidic X (PA-X) (22, 23). Treatment of IAV-infected cells with the eukaryotic translation initiation factor 4A (eIF4A) inhibitors pateamine A and silvestrol at early times postinfection induced SG formation and impeded IAV replication by blocking the accumulation of viral proteins and downstream viral genome replication (23, 24). However, eIF4A inhibitors triggered SG formation and cytotoxic effects in uninfected cells as well, limiting their potential utility as antivirals. Here, using an image-based high-content screen, we identified two FDA-approved thiopurine analogs that selectively triggered SG formation in IAV-infected cells. These thiopurines, 6-thioguanine (6-TG) and 6-thioguanosine (6-TGo), blocked IAV replication in a dose-dependent manner. Unlike eIF4A inhibitors, these molecules selectively disrupted the processing and accumulation of viral glycoproteins by activating the UPR. Our data suggest that UPR-inducing molecules could be effective host-targeted antivirals against viruses that depend on ER processes to support efficient replication. Induction of UPR by 6-TG and 6-TGo represents a novel host-directed antiviral mechanism and reveals a previously unrecognized unique mechanism of action that distinguishes them from the closely related thiopurine 6-mercaptopurine (6-MP) or nucleobase and nucleoside analogs like 5-fluorouracil (5-FU) and ribavirin.

## RESULTS

### Thiopurine analogs 6-TG and 6-TGo selectively induce SGs in IAV-infected cells.

**FIG 1:**
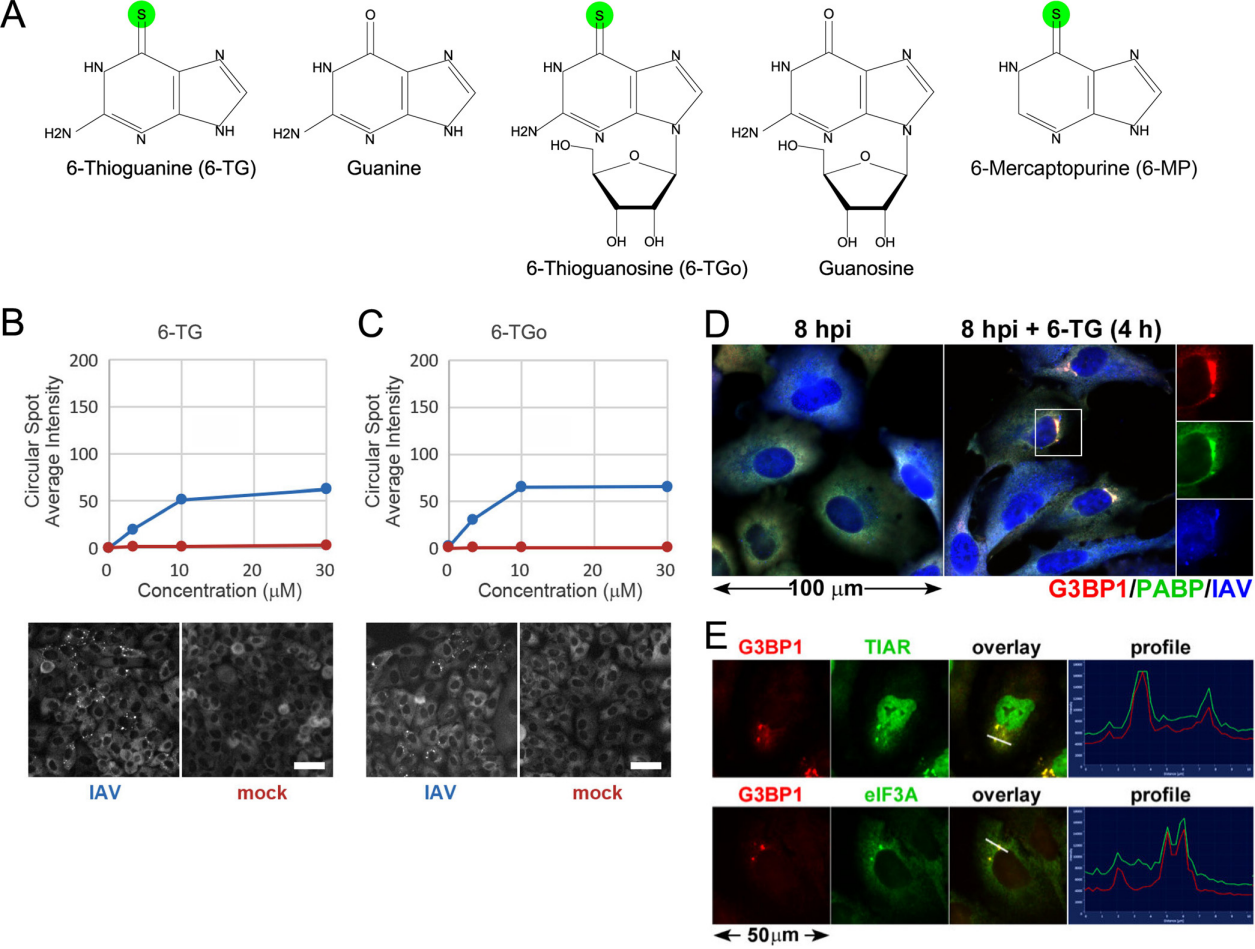
Thiopurine analogs 6-TG and 6-TGo selectively induce stress granules in IAV-infected cells. (A) Structures of 6-TG and 6-TGo compared to structurally similar nucleobases and nucleosides. Sulfur atoms in 6-TG, 6-TGo, and 6-MP are highlighted in green. (B and C, top) Circular spot intensity quantification of EGFP-G3BP focus formation in cells infected with IAV-Udorn (blue) or mock-infected cells (red) treated with increasing doses of 6-TG (B) and 6-TGo (C). (Bottom) Representative Cellomics images of the EGFP channel of cells treated with 30 μM 6-TG (B) and 6-TGo (C). (D) Immunofluorescence staining of A549 cells infected with IAV-CA/07 and treated with 6-TG or mock treated for 4 h. The stress granule markers G3BP1 (red) and PABP (green) colocalize in cytoplasmic stress granules (separate channels are shown in the outset). Infected cells are stained with anti-IAV antibody (blue). (E) Immunofluorescence staining of A549 cells treated as described above for panel D for the stress granule markers G3BP1 (red), TIAR (green) (top), and eIF3A (green) (bottom). White lines correspond to pixel intensity profiles shown on the right, as captured using Zeiss ZEN software.

To identify molecules that selectively induce antiviral SG formation in IAV-infected cells without inducing SGs in uninfected cells, we developed an image-based high-content screen. Clonal A549[EGFP-G3BP1] cells (22) stably expressing the core SG protein Ras-GAP SH3 domain binding protein 1 (G3BP1) fused to enhanced green fluorescent protein (EGFP) were selected because they display rapid and uniform SG formation in response to known SG-inducing treatments (e.g., sodium arsenite [As] or silvestrol). We screened >50,000 small molecules from the Prestwick, Sigma LOPAC, and Chembridge DiverSet collections for virus-specific SG induction and identified two thiopurines, 6-thioguanine (6-TG) and 6-thioguanosine (6-TGo), that triggered dose-dependent SG formation in IAV-infected cells (Fig. 1A to C). Specifically, SGs formed in approximately 10% of 6-TG-treated or 6-TGo-treated infected cells; no SGs were detected in mock-infected cells treated with either drug at the highest concentration (Fig. 1B and C). These findings were confirmed in parental A549 cells infected with IAV strain A/California/07/2009 (H1N1) (IAV-CA/07); 6-TG treated cells displayed the formation of foci that contained the SG constituent proteins G3BP1 and poly(A) binding protein (PABP) (Fig. 1D). These foci also contained the canonical SG proteins TIA related (TIAR) and eukaryotic translation initiation factor 3A (eIF3A) (Fig. 1E), supporting their identity as bona fide SGs.

### Thiopurine analogs inhibit IAV replication.

**FIG 2:**
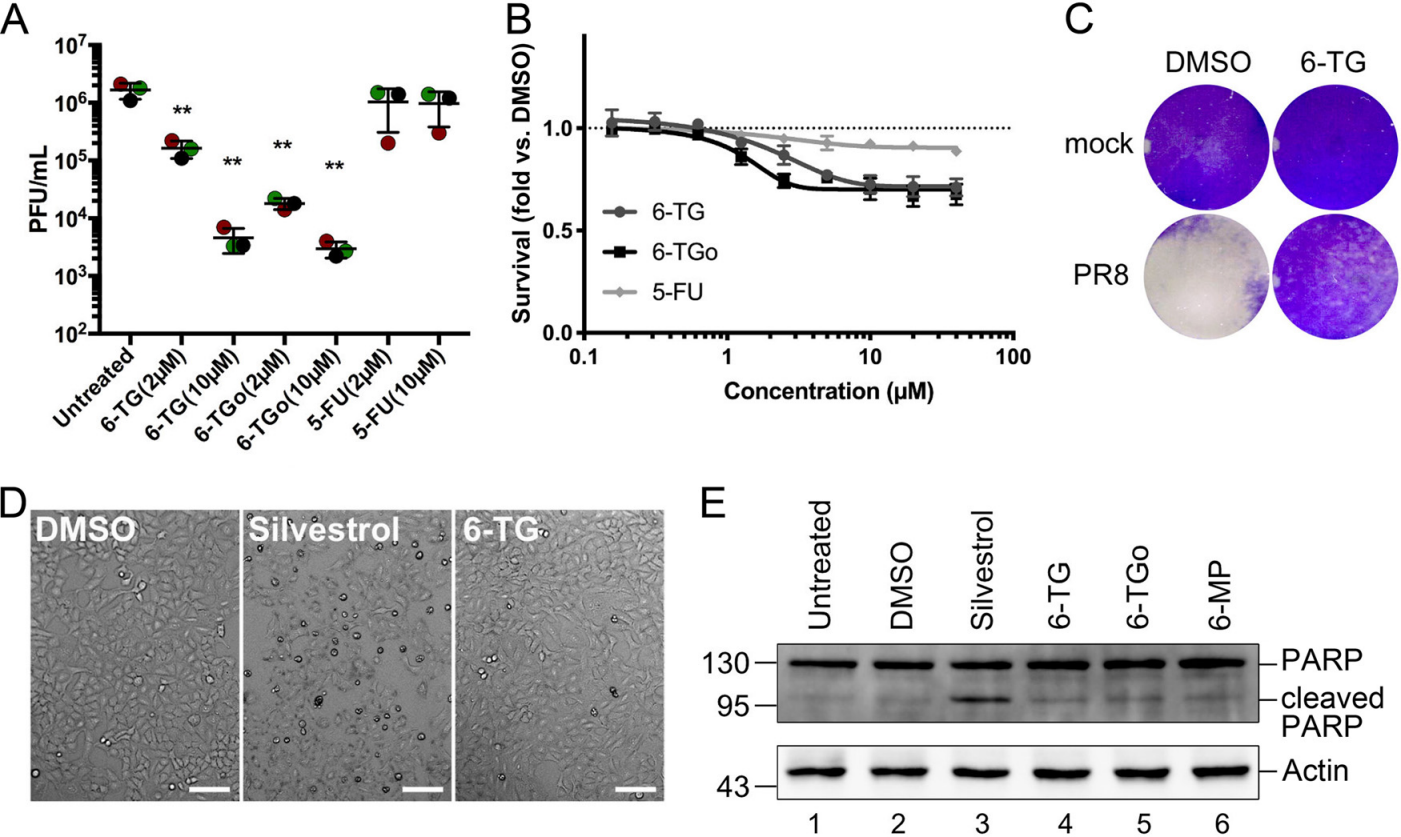
6-TG and 6-TGo inhibit IAV replication. (A) A549 cells were mock infected or infected with IAV-PR8 at an MOI of 0.1. After 1 h, cell monolayers were washed and overlaid with medium containing drugs at the indicated concentrations or the vehicle control. At 24 hpi, cell supernatants were collected, and infectious virions were enumerated by a plaque assay. Data from 3 independent experiments are graphed (*n* = 3), and error bars denote standard deviations. One-way ANOVA and Tukey’s *post hoc* multiple-comparison tests were done to determine statistical significance (**, *P* value of <0.01). (B) A549 cells were treated with increasing doses of 6-TG, 6-TGo, 5-FU, or the vehicle control (DMSO) for 23 h, and cell viability was measured using an alamarBlue assay. Relative fluorescence units were normalized to the value for the vehicle control. Error bars represent standard deviations (*n* = 3). (C) Vero cells were infected with IAV-PR8 and treated with 6-TG (10 μM) or the vehicle (DMSO). After 72 hpi, cells were fixed with 5% formaldehyde and stained with 1% crystal violet. (D) Representative phase-contrast images of A549 cell monolayers treated with silvestrol (320 nM), 6-TG (10 μM), or the vehicle (DMSO) control for 23 h. Bars, 100 μm. (E) Lysates of A549 cells treated with silvestrol (320 nM), the indicated thiopurines (10 μM), or the vehicle control (DMSO) were analyzed by Western blotting for total PARP (full length and cleaved). Actin was used as a loading control.

Next, we wanted to determine whether thiopurine-mediated SG induction indicated a disruption of viral replication. A549 cells were infected with IAV strain A/Puerto Rico/8/1934 (H1N1) (IAV-PR8) and treated with 6-TG, 6-TGo, or controls at 1 h post-infection (hpi). Cell supernatants were harvested at 24 hpi, and infectious virions were enumerated by a plaque assay. Despite SG induction in only a fraction of virus-infected cells, we observed a sharp dose-dependent decrease in virion production from thiopurine-treated cells; treatment with 2 μM 6-TG reduced virion production by ∼10-fold, whereas 2 μM 6-TGo reduced virion production by ∼100-fold (Fig. 2A). Furthermore, treatment of IAV-infected cells with 10 μM concentrations of either 6-TG or 6-TGo led to even greater inhibition of IAV production (Fig. 2A). By contrast, the nucleobase analog 5-fluorouracil (5-FU) had no effect on IAV replication at 2 μM and 10 μM doses. Using an alamarBlue assay, we observed a ∼30% reduction in A549 cell viability in the presence of 10 μM doses of 6-TG/6-TGo, compared to an ∼10% reduction with the highest doses of 5-FU (Fig. 2B). 6-TG treatment partially protected Vero cell monolayers from IAV-induced cell death over a 72-h incubation without overt cytotoxicity (Fig. 2C). Compared to the SG-inducing translation inhibitor silvestrol, which causes apoptosis in A549 cells upon prolonged exposure (25), we did not observe significant disruption of cell monolayers by 6-TG treatment (Fig. 2D) or induction of apoptosis as measured by poly(ADP-ribose) polymerase (PARP) cleavage (Fig. 2E). This is consistent with a recent report of 6-TG-mediated cytostatic rather than cytotoxic effects on A549 cells (26). Taken together, our data suggest that both 6-TG and 6-TGo elicit a broad dose-dependent antiviral effect against IAV that was not shared by the nucleobase analog 5-FU.

### 6-TG treatment inhibits HA and NA glycoprotein processing and accumulation without affecting viral transcription.

**FIG 3:**
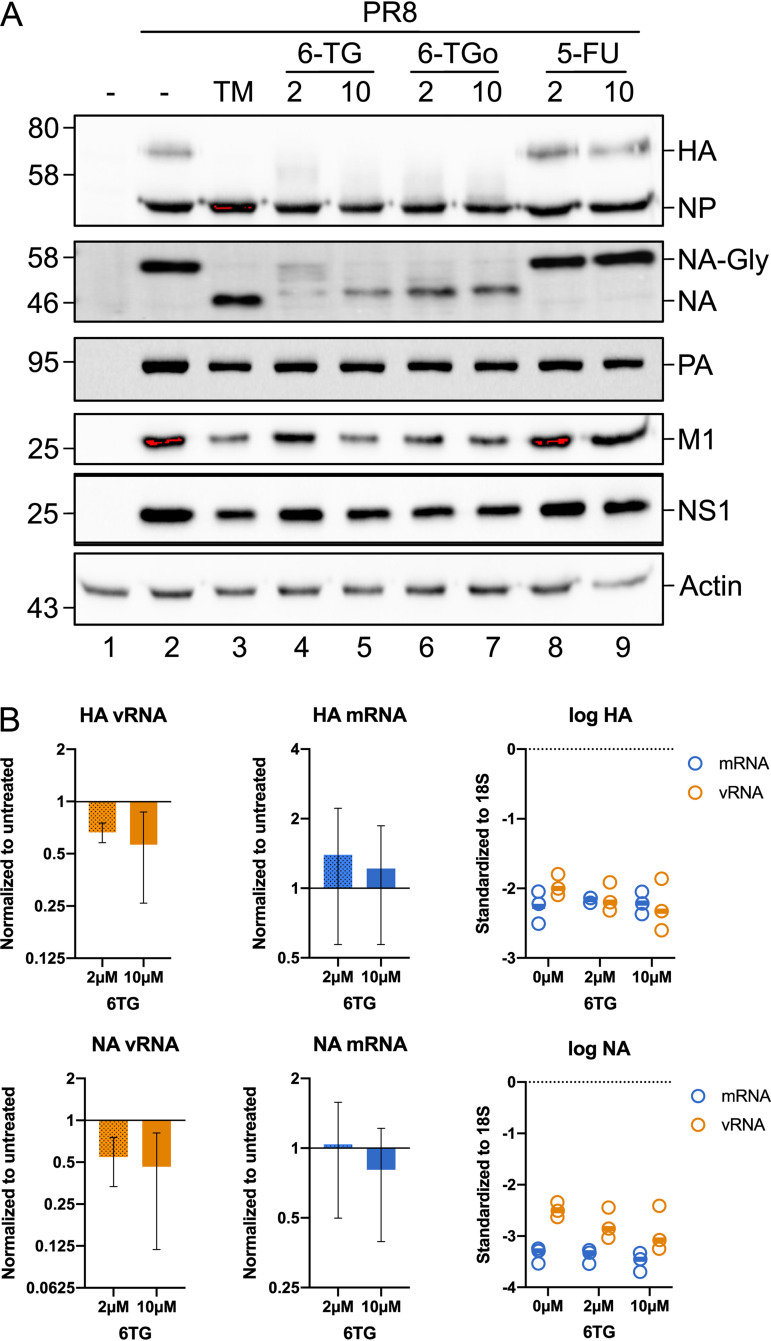
6-TG treatment inhibits HA and NA glycoprotein processing and accumulation without affecting transcript levels. (A) A549 cells were mock infected or infected with IAV-PR8 at an MOI of 0.1. After 1 h, cell monolayers were washed and overlaid with medium containing drugs at the indicated concentrations or the vehicle control (−). At 24 hpi, cell lysates were collected and analyzed by Western blotting using a polyclonal IAV antibody that detects HA, NP, and M1 as well as antibodies that detect IAV PA, NA, NS1, or cellular actin. A representative blot from 3 independent experiments is shown. (B) The levels of IAV HA and NA mRNAs, and HA and NA genomic vRNAs, were measured by RT-qPCR in A549 cells infected with IAV-PR8 and treated with 2 μM or 10 μM 6-TG or the vehicle control. Total RNA was isolated at 24 hpi. Changes in RNA levels were calculated by the ΔΔ*C_T_* method and normalized to 18S rRNA. Error bars represent standard deviations (*n* = 3). Circles represent biological replicates, and lines represent mean values.

Next, we compared the accumulation of viral proteins in cells treated with thiopurines and 5-FU (Fig. 3A). We previously observed that SG-inducing eIF4A inhibitors decreased the accumulation of all IAV proteins and blocked progression through the replication cycle by preventing the “switch” of the RNA-dependent RNA polymerase (RdRp) from transcription to genome replication (24). By contrast, treatment of IAV-PR8-infected cells with 6-TG and 6-TGo selectively blocked the processing and accumulation of HA and NA glycoproteins, similar to the effect of the N-linked glycosylation inhibitor tunicamycin (TM) (Fig. 3A). For NA, 6-TG and 6-TGo caused a dose-dependent accumulation of faster-migrating species, reminiscent of treatment with TM. For HA, 6-TG and 6-TGo likewise caused a dose-dependent decrease in the accumulation of glycosylated species, while the faster-migrating unglycosylated protein was difficult to visualize as it migrated to the same position as NP on immunoblots probed with polyclonal anti-IAV antibodies that detect NP, M1, and HA concurrently. While the accumulation of NP or polymerase acidic (PA) protein was not affected, matrix protein 1 (M1) and NS1 levels were moderately reduced in the presence of 6-TG and 6-TGo. This effect on M1 and NS1 accumulation was similar to that of TM treatment (Fig. 3A), which suggests that the synthesis and processing of viral glycoproteins, and the accumulation of M1 and NS1, may be linked in this infection model. 5-FU, which had no effect on viral replication over the 24-h time course in these cells (Fig. 2A), likewise had no effect on the accumulation of IAV proteins (Fig. 3A). Consistent with the notion of selective inhibition of IAV glycoprotein synthesis and maturation, we observed that 6-TG had no effect on the accumulation of IAV-PR8 *HA* or *NA* transcripts or the function of the RdRp in genome replication, as 6-TG had little effect on the accumulation of HA and NA genome segments (Fig. 3B). Taken together, these data support a novel mechanism of action for these SG-inducing thiopurine analogs in selectively inhibiting the processing and accumulation of IAV glycoproteins and impairing IAV replication.

### 6-TG and 6-TGo activate the UPR, and chemical mitigation of ER stress restores the synthesis of viral glycoproteins.

**FIG 4:**
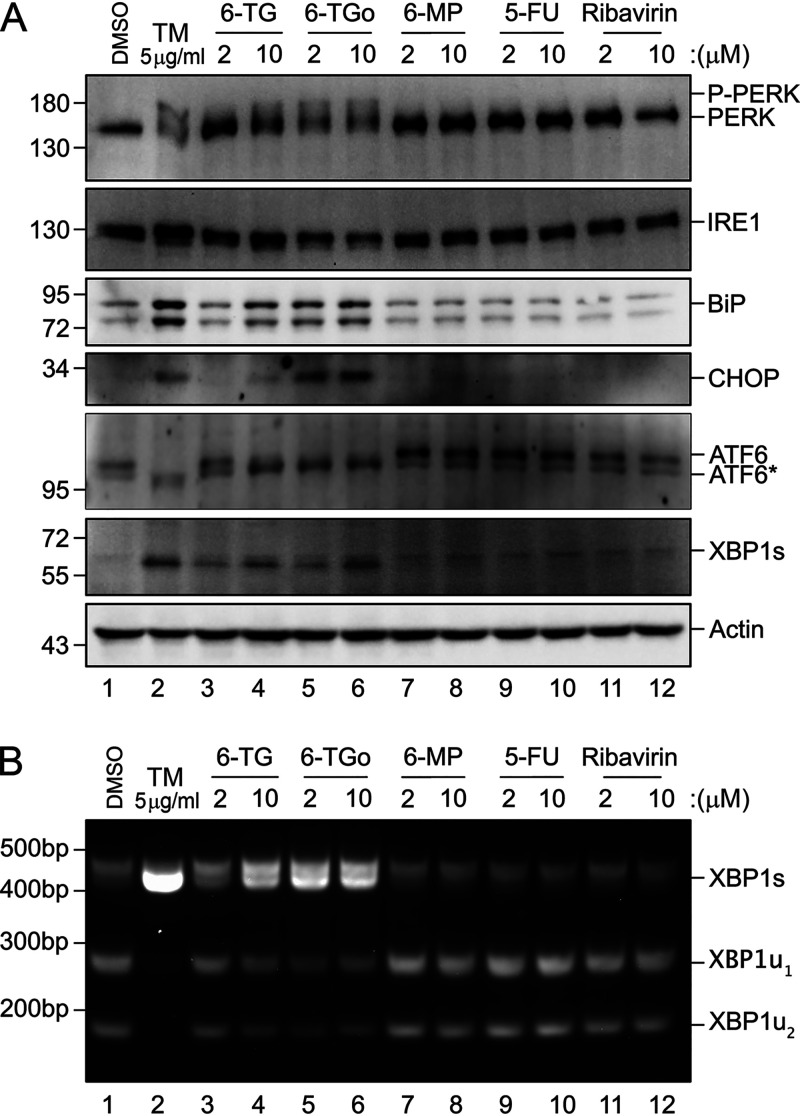
6-TG and 6-TGo activate the UPR. (A) A549 cells were treated with 6-thioguanine (6-TG), 6-thioguanosine (6-TGo), 6-mercaptopurine (6-MP), 5-fluorouracil (5-FU), or ribavirin at the indicated concentrations for 6 h prior to harvesting lysates for immunoblotting for the indicated cellular proteins. Tunicamycin (TM) at 5 μg/ml served as a positive control for UPR activation. ATF6* indicates a lower-molecular-weight species that is not cleaved to its active form. (B) cDNA was generated from total RNA that was isolated from treated cells. XBP1 mRNA splicing was determined by the semiquantitative RT-PCR splicing assay. XBP1u_1_ and XBP1u_2_ indicate the cleaved products from digesting the unspliced XBP1 cDNA with PstI.

By inhibiting N-linked glycosylation, TM treatment impairs the bulk processing of secreted and transmembrane proteins, which causes ER stress and UPR activation (27). Using TM, we confirmed that all three arms of the UPR (PERK, IRE1, and ATF6) are intact in the A549 cells that we use to model IAV infection. Specifically, TM treatment caused (i) PERK phosphorylation and accumulation of CHOP; (ii) IRE1 phosphorylation, X-box binding protein 1 (*XBP1*) mRNA splicing, and accumulation of XBP1s protein; and (iii) ATF6 activation and accumulation of BiP protein (Fig. 4). We observed that both 6-TG and 6-TGo activated all three arms of the UPR in a dose-dependent manner. The magnitude of these effects correlated well with our previous observations of 6-TG/6-TGo-mediated inhibition of IAV glycoprotein processing and accumulation (Fig. 3A) and replication (Fig. 2A), with 6-TGo displaying greater potency at a lower concentration than 6-TG. By contrast, the related thiopurine 6-mercaptopurine (6-MP), which differs from 6-TG only by the absence of an amino group (Fig. 1A), had no effect on the UPR (Fig. 4A and B). The nucleobase analog 5-FU and the nucleoside analog ribavirin also had no effect on the UPR in A549 cells. These data demonstrate that 6-TG and 6-TGo activate the UPR in A549 cells in a manner that correlates well with the negative impacts that we observed previously in our IAV infection model.

**FIG 5:**
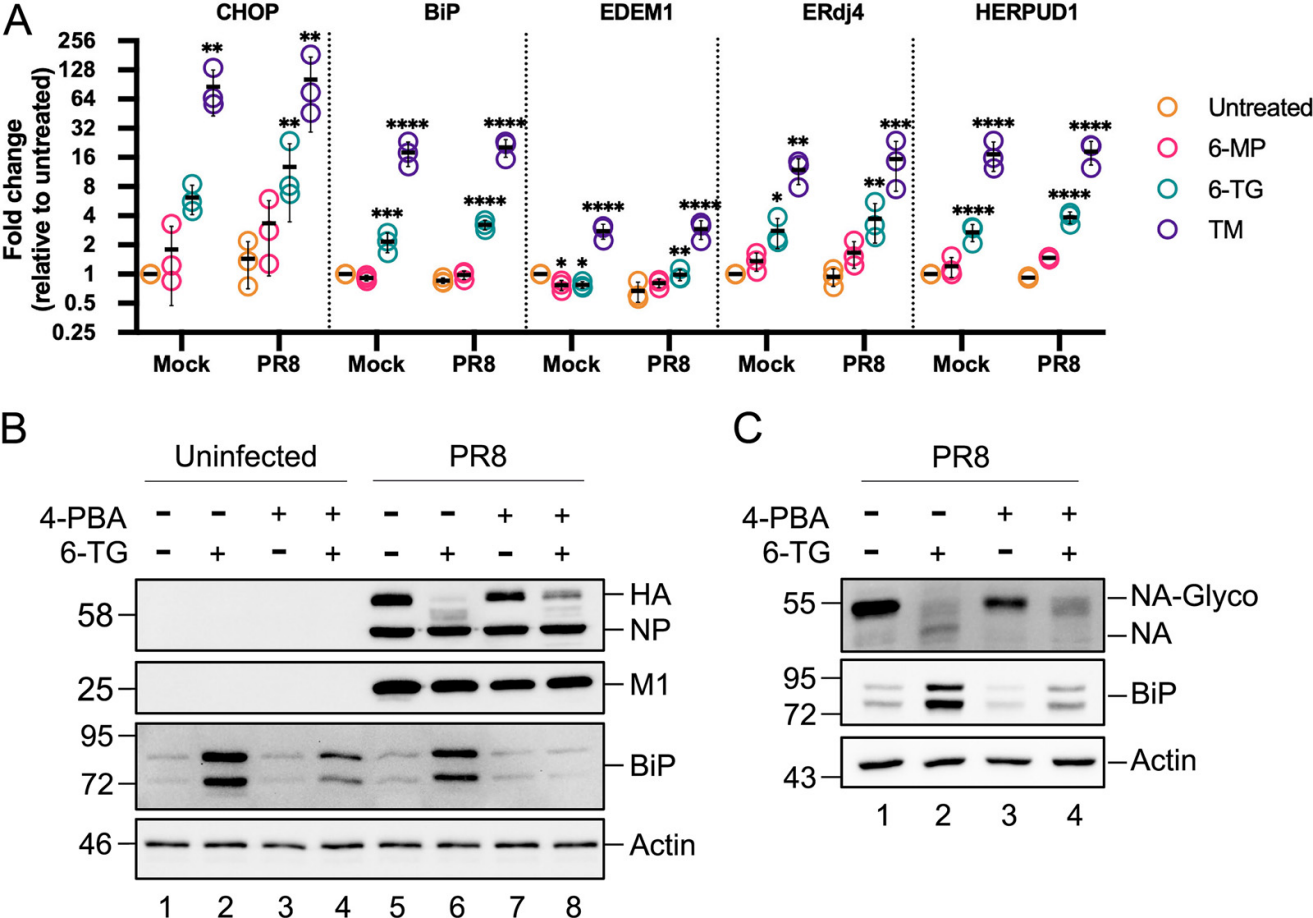
6-TG treatment upregulates UPR genes, and chemical chaperones can limit the UPR and restore viral glycoprotein accumulation. (A) A549 cells were infected with IAV-PR8 at an MOI of 1, washed, and overlaid with medium containing 6-MP, 6-TG, or TM. Cell lysates were collected at 24 hpi, and RNA was isolated and processed for RT-qPCR. Changes in CHOP, BiP, EDEM1, ERdj4, and HERPUD1 mRNA levels were calculated by the ΔΔ*C_T_* method, normalized using 18S rRNA as a reference gene, and standardized to mock. Error bars represent standard deviations (*n* = 3). Circles represent individual replicates, and lines represent mean values. Statistical significance was calculated via two-way ANOVA followed by a Dunnett multiple-comparison test. (B and C) A549 cells were mock infected or infected with IAV-PR8 at an MOI of 1. After 1 h, cells were washed and incubated with 20 μM 6-TG or the vehicle control, with or without 10 μM 4-PBA. At 20 hpi, cell lysates were harvested and probed using a polyclonal IAV antibody that detects IAV HA, NP, and M1 proteins (B); IAV NA (C); and antibodies to cellular BiP and actin. Representative data from 3 independent experiments are shown.

To corroborate our observation of thiopurine-mediated UPR activation and extend it to IAV-infected cells, A549 cells were mock infected or IAV-PR8 infected and treated with 6-TG, 6-MP, or TM for 23 h before harvesting RNA for reverse transcription-quantitative PCR (RT-qPCR) analysis of UPR gene expression. We analyzed transcripts produced from target genes linked to each arm of the UPR: the ATF4 target gene *CHOP*, XBP1s target genes *EDEM1* and *ERdj4*, and ATF6 target genes *BiP* and *HERPUD1*. As expected, TM treatment caused strong induction of all 3 arms of the UPR and increased the transcription of all five target genes in mock-infected cells and infected cells alike (Fig. 5A). This strong and consistent transcriptional output between mock-infected and infected cells suggests that the UPR remains largely intact during IAV-PR8 infection of A549 cells. Treatment with 6-MP had little effect on UPR gene expression in mock-infected or infected cells (Fig. 5A), consistent with our previous observations (Fig. 4). By contrast, 6-TG treatment caused statistically significant increases in transcription from all five UPR target genes, with a trend toward an increased output in IAV-PR8-infected cells compared to mock-infected cells (Fig. 5A). These observations confirm that 6-TG activates all three arms of the UPR, whereas the chemically similar thiopurine 6-MP does not.

UPR activation can be mitigated by chemical chaperones like 4-phenylbutyrate (4-PBA) that have been shown to restore the proper folding and trafficking of misfolded mutant proteins like the Δ508 allele of the cystic fibrosis transmembrane conductance regulator (Δ508-CFTR) (28). Here, we used 4-PBA to determine whether UPR activation was required to disrupt IAV glycoprotein synthesis and processing. In mock-infected A549 cells, 6-TG treatment caused a strong accumulation of BiP, which could be partially reversed by the co-administration of 4-PBA (Fig. 5B). As we demonstrate above, 6-TG treatment of IAV-infected cells disrupted the processing and accumulation of HA and NA (Fig. 5B and C), whereas there was little change in the accumulation of NP and M1 (Fig. 5B). In contrast, the coadministration of 6-TG and the chemical chaperone 4-PBA diminished the accumulation of BiP and partially restored HA and NA synthesis and processing in infected cells (as inferred by electrophoretic mobility). This suggests that the 6-TG-mediated activation of the UPR is at least partially responsible for the diminished accumulation of IAV glycoproteins in infected cells.

### 6-TG and 6-TGo have little impact on host cell protein synthesis.

**FIG 6:**
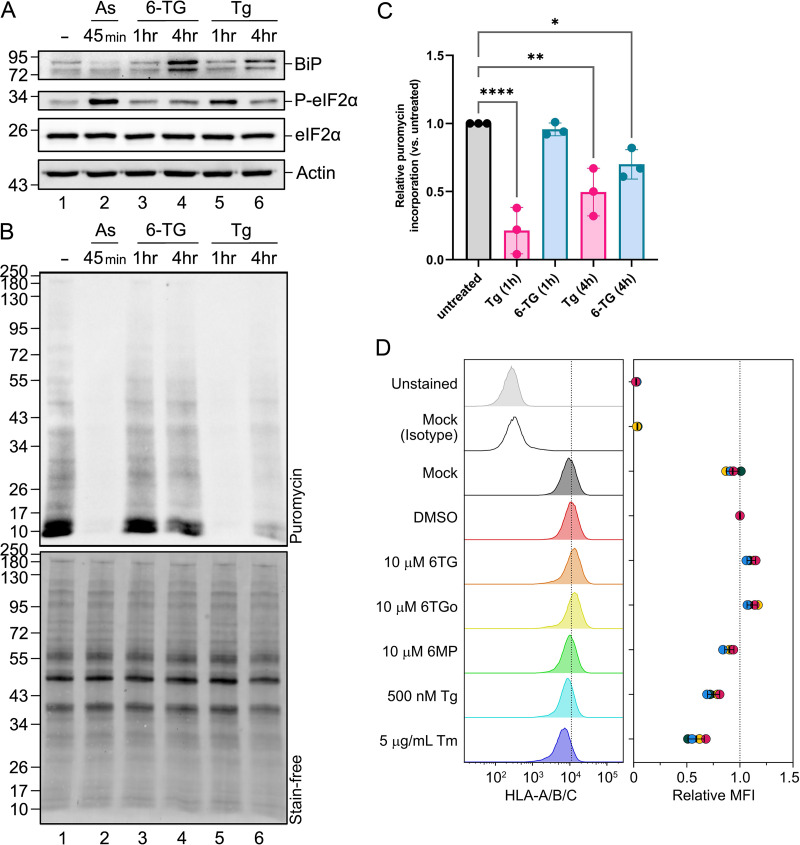
6-TG treatment does not block global translation or decrease surface expression of human leukocyte antigen A/B/C (HLA-A/B/C) glycoproteins. (A) A549 cells were treated with 10 μM 6-TG and 1 μM thapsigargin (Tg) for the indicated times or with 500 μM sodium arsenite (As) for 45 min prior to harvesting cell lysates and Western blotting for BiP, eIF2α, phosphorylated eIF2α, or actin. (B) Protein synthesis in cells treated as described above for panel A was analyzed using a puromycylation assay. (C) The puromycin signal (B, top) was quantified and normalized to total protein loading (B, bottom, Stain-free) from 3 independent replicates. Error bars indicate standard deviations. One-way ANOVA and Dunnett multiple-comparison tests were done to determine statistical significance (*, *P* value of <0.05; **, *P* value of <0.01; ***, *P* value of <0.001). (D) A549 cells were treated as indicated, and the surface expression of HLA-A/B/C was analyzed using flow cytometry. The histograms from one representative experiment are depicted on the left, where the vertical dotted line indicates the median fluorescence intensity (MFI) of HLA-A/B/C in the DMSO-treated sample. The histograms on the right display the MFI of HLA-A/B/C on the cell surface for each treatment shown, with the individual data points (colored circles) with black lines indicating the means ± standard deviations (*n* = 4, except for the isotype control [*n* = 2]). The vertical dotted line in the graph indicates the mean MFI from the DMSO-treated samples.

A common feature of the UPR is PERK-mediated phosphorylation of eIF2α and activation of the ISR that coincides with the arrest of global protein synthesis. Because 6-TG and 6-TGo activate all three arms of the UPR, including PERK, we sought to understand whether these thiopurines affect global protein synthesis via ISR activation. To test this directly, A549 cells were treated for 1 h or 4 h with 6-TG or the positive control thapsigargin (Tg) that activates PERK by depleting ER calcium stores required to support protein folding. Sodium arsenite (As) was also included as a positive control that triggers HRI-dependent eIF2α phosphorylation and ISR activation without engaging the UPR (29). As or Tg treatment for 1 h increased eIF2α phosphorylation, whereas after 4 h of Tg treatment, eIF2α phosphorylation had diminished from its peak, as expected, and BiP accumulation was evident, consistent with UPR activation (Fig. 6A). A 4-h 6-TG treatment also increased eIF2α phosphorylation but not to the same extent as Tg. 6-TG also caused a strong increase in BiP accumulation, likely reflecting the activation of all three arms of the UPR, two of which (PERK and ATF6) directly regulate BiP levels. To further investigate the effects of 6-TG on the ISR, a parallel experiment was conducted with the same drug treatments accompanied by a 10-min pulse of puromycin to label newly synthesized proteins (30, 31). As expected, consistent with our previous observations of strong eIF2α phosphorylation (Fig. 6A), we observed that treatment with As or Tg for 1 h dramatically reduced new protein synthesis without affecting total cellular protein content (Fig. 6B and C). By 4 h after treatment with Tg, new protein synthesis was partially restored, consistent with entry into the resolution phase of the ISR and dephosphorylation of eIF2α (Fig. 6A to C). Finally, despite clear evidence of UPR activation and BiP upregulation (Fig. 6A), 6-TG only modestly inhibited the global synthesis of new cellular proteins (Fig. 6B and C).

Approximately 40% of all cellular proteins are made in the ER (32). Since 6-TG and 6-TGo selectively inhibited the synthesis and processing of IAV HA and NA glycoproteins, we reasoned that cellular glycoproteins might also be sensitive to these drugs. We elected to study the effects of thiopurines on human leukocyte antigen A/B/C (HLA-A/B/C) synthesis and maturation, as it is a very well-studied type I transmembrane glycoprotein that traverses the secretory pathway to access the cell surface (33). A549 cells were treated with thiopurine drugs or controls for 23 h prior to harvest, surface staining with anti-HLA-A/B/C antibody, and flow cytometric analysis. As expected, HLA expression was clearly inhibited by treatment with the ER stress-inducing drugs Tm and Tg, which would be expected to interfere with glycosylation and protein trafficking (Fig. 6D). In contrast, thiopurine treatment had very little effect on surface HLA-A/B/C expression; the negative-control thiopurine 6-MP did not affect surface HLA-A/B/C presentation, whereas the levels of surface HLA-A/B/C were slightly increased following treatment with 6-TG and 6-TGo. This is consistent with established mechanistic links between moderate ER stress and surface HLA-A/B/C expression, which have been studied extensively in the context of innate immune recognition by natural killer (NK) cells (34). Thus, we have established that 6-TG and 6-TGo have very little impact on the rates of global new protein synthesis as well as the synthesis and trafficking of a model host cell glycoprotein despite strongly blocking the accumulation of IAV HA and NA proteins.

### Inhibition of the integrated stress response does not restore NA processing and oligomerization in 6-TG-treated cells.

**FIG 7:**
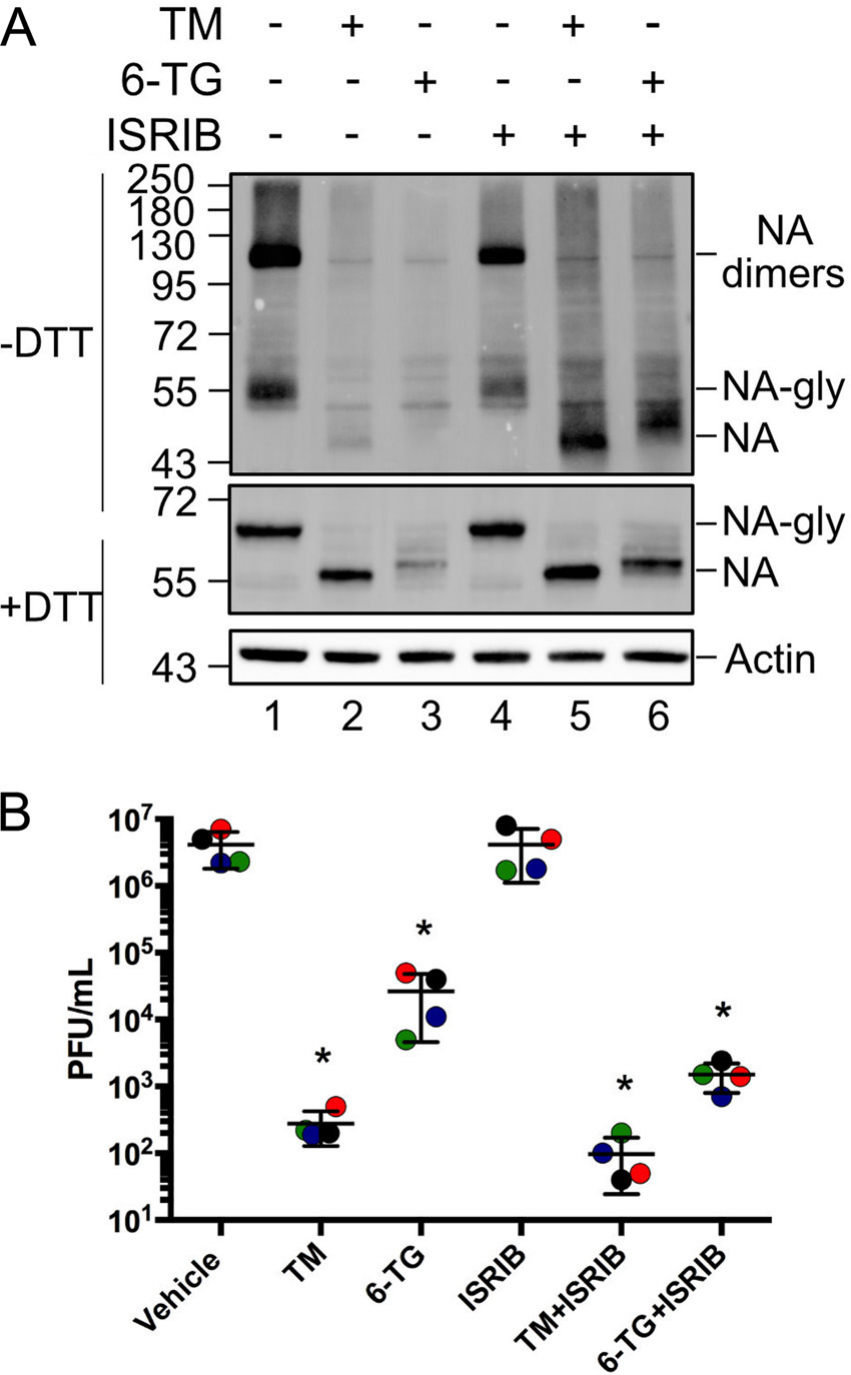
Integrated stress response inhibition restores NA synthesis in the presence of 6-TG, but NA processing and virion production remain impaired. A549 cells were infected with IAV-PR8 at an MOI of 1. After 1 h, cells were washed and treated with tunicamycin (TM) (5 μg/ml), 6-thioguanine (6-TG) (10 μM), and/or 500 ng/ml ISRIB. (A) At 24 hpi, cell lysates were collected and processed for native SDS-PAGE and immunoblotting using an anti-NA antibody. N-glycosylated forms of NA are indicated as NA-gly, whereas glycosylated NA dimers are indicated as dimers. Actin was used as a loading control. (B) At 24 hpi, cell supernatants were collected, and infectious IAV-PR8 virions were enumerated by a plaque assay. Error bars represent the standard deviations between biological replicates (*n* = 4). Circles represent biological replicates, and lines represent mean values.

IAV glycoproteins are translocated into the ER, where they are modified with N-linked glycans and organize into oligomeric complexes. Upon synthesis in the ER, the type II transmembrane protein NA is glycosylated and forms dimers linked by intermolecular disulfide bonds in the stalk region (13) that then assemble into tetramers (35). We investigated the effect of 6-TG on NA processing and oligomerization using sodium dodecyl sulfate (SDS)-PAGE/immunoblotting procedures in the presence or absence of the disulfide bond-reducing agent dithiothreitol (DTT). Since NA tetramers are known to dissociate into dimers during electrophoresis (12, 36), we annotated the ∼120-kDa band as dimers (Fig. 7A). We observed intact glycosylated NA dimers and monomers in mock-treated IAV-PR8-infected cells, which were resolved into ∼60-kDa glycosylated NA monomers in the presence of DTT. Unglycosylated NA monomers were not detected in mock-treated cells at steady state, indicating that N-glycosylation is a rapid initial step in NA processing in the ER. TM treatment eliminated NA dimers, leaving a minor fraction of unglycosylated NA monomers. 6-TG treatment diminished the accumulation of all forms of NA, yielding a distinct residual band that migrated closer to the size of the unglycosylated NA monomers from TM-treated cells; this suggests that 6-TG treatment interferes with proper N-glycosylation of nascent NA proteins. Treatment with integrated stress response inhibitor (ISRIB), which prevents ISR-mediated translation arrest by maintaining eIF2B activity (37, 38), rescued the accumulation of NA monomers in both TM- and 6-TG-treated cells. However, ISRIB was not able to restore NA glycosylation and oligomerization. These data provide further evidence that 6-TG inhibits IAV glycoprotein accumulation via UPR/ISR activation and extend our understanding by demonstrating that ISR suppression does not fully reverse these effects. This is further supported by our observations that the administration of ISRIB alone had no impact on IAV replication, while the co-administration of ISRIB with TM- or 6-TG failed to restore virion production in single-cycle infection assays (Fig. 7B).

### Genetic deletion of PERK potentiates the 6-TG-mediated inhibition of influenza A virus replication.

**FIG 8:**
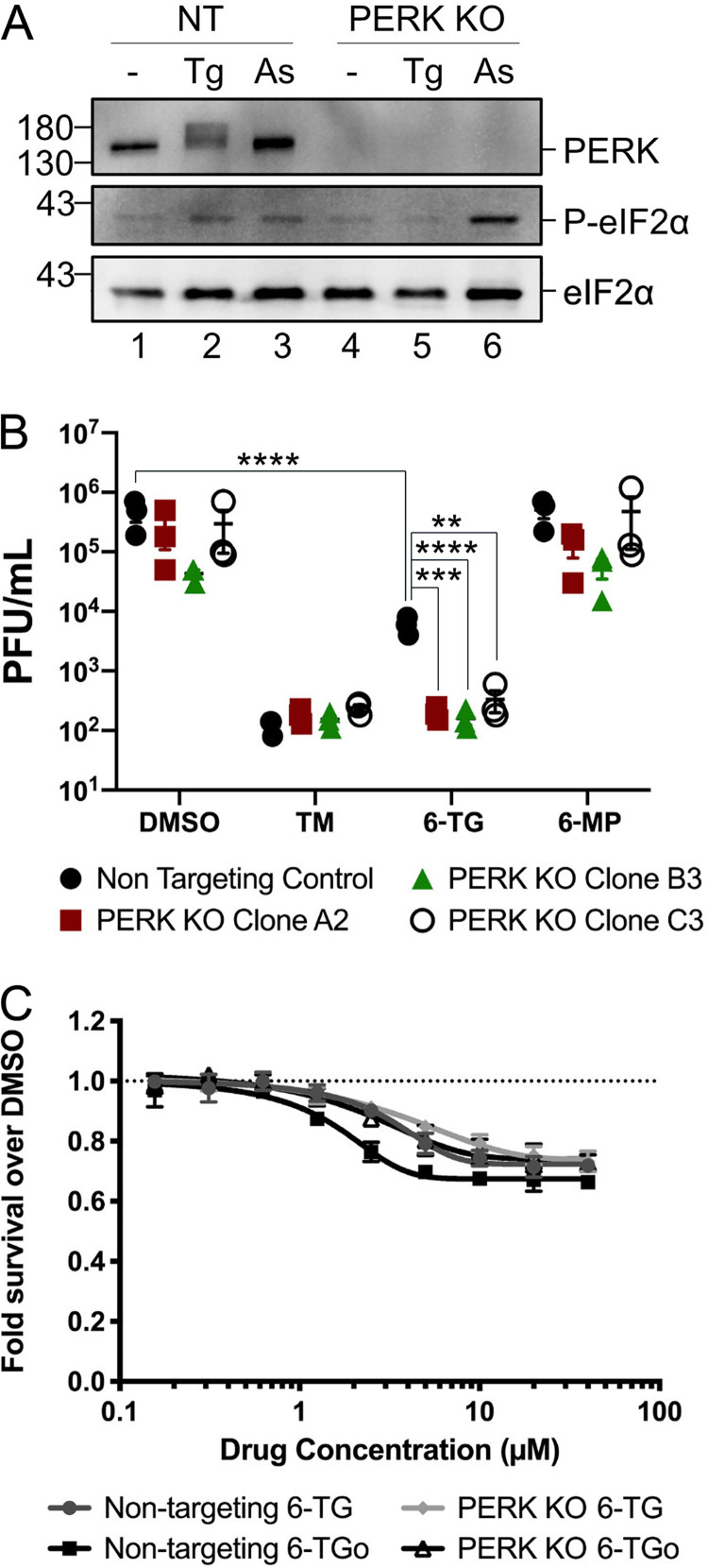
Genetic deletion of PERK enhances the antiviral effect of 6-TG. (A) Western blotting of PERK KO cells (clone B3) and nontargeting control gRNA lentivirus-transduced cells (NT) treated with 1 μM thapsigargin (Tg) or 500 μM sodium arsenite (As). Lysates were collected at 1 h post-treatment and analyzed for PERK expression and activation and total and phosphorylated eIF2α. (B) A549 PERK KO cells (clones A2, B3, and C3) and the nontargeting control cell line were infected with PR8 at an MOI of 0.1 and treated with tunicamycin (TM) (5 μg/ml), 6-thioguanine (6-TG) (10 μM), and 6-mercaptopurine (6-MP) (10 μM) for 23 h. The supernatant was collected at 24 hpi, and viral progeny were quantified by plaque assay. Statistical significance was calculated via two-way ANOVA followed by a Dunnett multiple-comparison test (only a subset of statistically significant differences is highlighted with asterisks for clarity). (C) A549 PERK KO clone B3 and nontargeting control cells were treated with escalating doses of 6-TG, 6-TGo, or the vehicle control for 23 h, and cell viability was measured using an alamarBlue assay. Relative fluorescence units were normalized to the vehicle control. Error bars represent standard deviations (*n* = 3).

Our observations that 6-TG activates PERK as part of a broader UPR (Fig. 4 and 5) and that ISRIB treatment partially restored NA protein accumulation in the presence of 6-TG but failed to rescue proper NA processing and IAV replication (Fig. 7A and B) motivated further investigation of the PERK arm of the UPR. To complement our pharmacological studies, we deleted *PERK* in A549 cells using CRISPR technology and assessed effects on IAV replication. Treatment of PERK knockout (KO) A549 cells and control A549 cells with arsenite for 1 h increased HRI-dependent eIF2α phosphorylation, whereas Tg treatment for 1 h could elicit PERK activation and eIF2α phosphorylation only in the control A549 cells (Fig. 8A). To investigate the role of PERK in this system, PERK knockout A549 cells and control cells were infected with IAV and treated with the dimethyl sulfoxide (DMSO) vehicle control, TM, 6-TG, or 6-MP for 23 h prior to harvesting progeny virions. As described above, inhibition of N-glycosylation with TM treatment diminished virion production by >1,000-fold in the control A549 cells, consistent with the important role of glycosylation in IAV envelope protein trafficking and virion production (39, 40), and a similar magnitude of inhibition was evident in PERK knockout A549 cells (Fig. 8B). Treatment with the negative-control thiopurine 6-MP had no significant impact on virion production in either the control or PERK knockout A549 cells. However, the deletion of PERK had a strong effect on virion production in the presence of 6-TG; treatment of control A549 cells with 6-TG caused an ∼1.5-log decrease in virion production, which increased to a ∼3-log reduction in the PERK knockout A549 cells. alamarBlue cell viability assays indicated that the reduction in virion production was not due to a cytotoxic effect of 6-TG or 6-TGo on PERK KO cells, as the only appreciable cytotoxic or cytostatic effect was observed in the control A549 cells with intact PERK following treatment with 6-TGo (Fig. 8C).

## DISCUSSION

Compared to current direct-acting antiviral drugs, effective host-targeted antivirals may provide a higher barrier to the emergence of drug-resistant viruses. However, it remains challenging to identify cellular pathways that can be targeted to disrupt viral replication without causing adverse effects on bystander uninfected cells. Here, we report that two chemically similar FDA-approved thiopurine analogs, 6-TG and 6-TGo, have antiviral effects that result from UPR activation and disruption of IAV glycoprotein synthesis and maturation. Importantly, our data demonstrate for the first time that 6-TG and 6-TGo are effective antivirals against influenza virus. 6-TG is currently used in clinical settings to treat acute lymphoblastic leukemia and other hematological malignancies, with the main mechanism of action involving conversion into thioguanine deoxynucleotides and subsequent incorporation into cellular DNA, which preferentially kills rapidly dividing cancer cells (21, 41). Furthermore, the active 6-TG metabolite 6-thioguanosine 5′-triphosphate was shown to inhibit the small GTPase Rac1 (42), which is believed to be largely responsible for the anti-inflammatory effects of 6-TG in treating inflammatory bowel disease (IBD) (43). Finally, 6-TG and the closely related thiopurine 6-mercaptopurine (6-MP) were demonstrated to have *in vitro* efficacy against coronaviruses as reversible, slow-binding inhibitors of the papain-like cysteine protease PL(pro) (25, 44, 45). Here, we demonstrate for the first time that 6-TG and 6-TGo, but not 6-MP or other nucleobase and nucleoside analogs (5-FU and ribavirin), rapidly induce the UPR in human cell lines. The selective disruption of viral glycoprotein accumulation in IAV-infected cells with minimal effects on other viral proteins suggests that UPR induction by 6-TG and 6-TGo is the main antiviral mechanism. Indeed, chemical chaperones and the ISR inhibitor ISRIB partially restored IAV HA and NA protein accumulation in cells treated with 6-TG. IAV replicates in the nucleus of infected cells, and the depletion of viral envelope glycoproteins that block infectious virion production minimally affects the synthesis of viral nucleic acids.

Our analyses of IAV-infected cells did not detect induction of UPR when high levels of the viral glycoproteins HA and NA accumulated at later times postinfection. Many viruses have mechanisms to interfere with different arms of the UPR, presumably to maximize the production and trafficking of viral glycoproteins, and IAV was previously shown to inhibit the activation of PERK and ATF6 (17, 18). Downstream, IAV blocks new host mRNA processing and protein synthesis through host shutoff mechanisms mediated by viral NS1 and polymerase acidic X (PA-X) proteins (46). Despite these viral mechanisms of suppression of host stress responses, 6-TG treatment elicited similar levels of UPR in both infected and uninfected cells, as measured by the transcriptional induction of genes downstream of all three arms of the UPR and the accumulation of BiP protein. Thus, in our system, it appears that IAV is vulnerable to UPR activation.

Despite multiple mechanisms deployed by IAV to block SG formation, 6-TG and 6-TGo treatment induced SGs in infected cells, which allowed us to identify these molecules in our image-based screen. We previously reported that in A549 cells, IAV inhibited SG formation triggered by treatment with Tg, a potent inducer of ER stress, with only 9% of infected cells forming SGs, compared to 35% of mock-infected cells (23). Thus, the induction of SGs in approximately 10% of infected cells by 6-TG and 6-TGo treatment appears consistent with our previous observations. However, unlike Tg, 6-TG and 6-TGo did not trigger SG formation in uninfected cells. The levels of UPR induction by these drugs were similar between infected and uninfected cells, suggesting that in IAV-infected cells, SG formation may not be triggered downstream of ER stress and PERK activation and may only partially contribute to the antiviral effects of thiopurines. Indeed, SGs formed in a fraction of infected cells, while the accumulation of the viral glycoproteins HA and NA was nearly completely blocked by 6-TG and 6-TGo.

What is the mechanism of UPR induction by 6-TG and 6-TGo? Our results suggest that the effects are unlikely to be mediated through DNA or RNA incorporation of 6-thioguanosine triphosphate because (i) the UPR was rapidly induced following a brief few hours of treatment; (ii) replicative stress does not specifically induce UPR; (iii) among viral proteins, only glycoprotein accumulation and processing were disrupted; and (iv) mRNA levels of HA and NA were not affected. Furthermore, the closely related thiopurine 6-MP that is also converted into 6-thioguanine triphosphate and is incorporated into nucleic acids did not induce the UPR and had no effect on IAV. Another nucleobase analog, 5-FU, which is also incorporated into nucleic acids and can even trigger SG formation upon a prolonged 48-h incubation (47), was similarly inactive in our assays. The second previously described antiviral mechanism of action of 6-TG and 6-MP that involves direct inhibition of viral cysteine proteases is similarly unlikely to have a major contribution in these experiments because UPR induction was triggered in both infected and uninfected cells and because, as mentioned above, 6-MP was not active in our assays. Thus, by process of elimination, we speculate that the mechanism of UPR induction by 6-TG and 6-TGo could involve GTPase inhibition. Numerous GTPases regulate ER homeostasis, including Rab GTPases that govern vesicular trafficking events and dynamin-like GTPases that regulate homotypic ER membrane fusion events required for the maintenance of branched tubular networks (48). Future studies will focus on identifying specific molecular targets of these UPR-inducing thiopurines using orthogonal biochemical and genetic screens.

## MATERIALS AND METHODS

### Cell lines.

Human lung adenocarcinoma A549 cells and Madin-Darby canine kidney (MDCK) cells were maintained in Dulbecco’s modified Eagle’s medium (DMEM; Thermo Fisher Scientific, Ottawa, ON, Canada) supplemented with 10% fetal bovine serum (FBS; Thermo Fisher Scientific, Grand Island, NY, USA) and 100 U/ml penicillin–100 μg/ml streptomycin–20 μg/ml glutamine (Pen/Strep/Gln; Wisent Bioproducts, St-Bruno, QC, Canada) at 37°C in a 5% CO_2_ atmosphere. All cell lines were purchased from the American Type Culture Collection (ATCC). The generation of A549[EGFP-G3BP1] cells was described previously (22).

### Influenza viruses and infections.

Viruses used in this study include A/Puerto Rico/8/34 (H1N1) (IAV-PR8), A/Udorn/1972 (H3N2) (IAV-Udorn), and A/California/07/2009 (H1N1) (IAV-CA/07). IAV-PR8 stocks were generated using the 8-plasmid reverse genetic system provided by Richard Webby (St. Jude Children’s Research Hospital, Memphis, TN, USA); IAV-Udorn was rescued from the 12-plasmid system provided by Yoshihiro Kawaoka (University of Wisconsin—Madison, Madison, WI, USA); IAV-CA/07 was provided by the Public Health Agency of Canada (PHAC) National Microbiology Laboratory. Stocks of influenza viruses were propagated in MDCK cells in IAV infection medium (DMEM supplemented with 0.5% bovine serum albumin [BSA], 20 μg/ml glutamine, and 1 μg/ml *N*-tosyl-l-phenylalanine chloromethyl ketone [TPCK]-treated trypsin). For infection, virus inocula were diluted in IAV infection medium without trypsin and added to cells for 1 h at 37°C. After inocula were removed, cell monolayers were washed with phosphate-buffered saline (PBS), and fresh IAV infection medium was added. Supernatants were harvested at 24 h postinfection (hpi) unless indicated otherwise, and the supernatant was incubated with TPCK-treated trypsin at 1.5 μg/ml for 1 h at 37°C to activate influenza virus HA. Plaque assays were performed in MDCK cells using 1.2% Avicel (FMC, Philadelphia, PA) overlays as described previously by Matrosovich et al. (49).

### Cellomics drug screen.

A549[EGFP-G3BP1] cells stably expressing EGFP-tagged G3BP1 (22) were seeded at 50,000 cells/well in 96-well optical-bottom plates in a 50-μl volume of 10% FBS-containing DMEM 18 h prior to infection. Cells were infected with IAV-Udorn (H3N2) at a multiplicity of infection (MOI) of 1.0 by the direct addition of 50 μl/well of the virus inoculum prediluted in 0.5% BSA-containing DMEM. Cells were treated with a small-molecule library (∼50,000 molecules) at 4 hpi using a pinning robot. At 8 hpi (4 h after drug treatment), cells were washed with PBS before fixation with 3% paraformaldehyde (PFA) in PBS with 500 ng/ml Hoechst 33342 for 20 min, followed by one more PBS wash. Automated image capture was performed using a Cellomics Arrayscan VTI HCS reader. The punctate EGFP-G3BP1 signal was acquired as “circ spot average intensity” using the compartment analysis algorithm. Every plate contained uninfected, untreated cells for the negative control and cells treated with silvestrol (300 nM) for the positive control of SG induction. Candidate hits that were initially identified in the screen were followed up by treating both mock- and IAV-Udorn-infected A549[EGFP-G3BP1] cells and analyzing G3BP1 puncta.

### Chemical inhibitors.

6-Thioguanine (6-TG), 2-amino-6-mercaptopurine riboside hydrate (6-TGo), 6-mercaptopurine (6-MP), 5-fluorouracil (5-FU), ribavirin, integrated stress response inhibitor (ISRIB), 4-phenylbutyric acid (4-PBA), tunicamycin (TM), sodium arsenite (As), and thapsigargin (Tg) (all obtained from Sigma-Aldrich Canada Co., Oakville, ON, Canada) were solubilized in dimethyl sulfoxide (DMSO) (with the exception of As, which was dissolved in water) and stored at −80°C. Stock concentrations were diluted to the indicated concentrations in medium.

### Cytotoxicity assay.

A549 cells were seeded at 10,000 cells/well in a 96-well plate. Drugs were diluted in infection medium (0.5% BSA-containing DMEM) at the indicated concentrations and incubated with the cells for 24 h. At 20 h posttreatment, 10% alamarBlue cell viability reagent (catalog number DAL1025; Thermo Fisher) was added to the cell monolayer, followed by 4 additional hours of incubation. Plates were analyzed on a FLUOstar Omega 96-well plate reader at an excitation wavelength of 544 nm and an emission wavelength of 580 to 590 nm. The relative fluorescence units for both the vehicle (DMSO) and drug treatments were first normalized to untreated cells. Drug treatments were then normalized to DMSO.

### Immunoblotting.

Cell monolayers were washed once with ice-cold PBS and lysed in 2× Laemmli buffer (4% [wt/vol] sodium dodecyl sulfate [SDS], 20% [vol/vol] glycerol, 120 mM Tris-HCl [pH 6.8]). DNA was sheared by repeated passage through a 21-gauge needle before 100 mM dithiothreitol (DTT) addition and boiling at 95°C for 5 min. Samples were stored at −20°C until analysis. The total protein concentration was determined by a DC protein assay (Bio-Rad), and equal quantities were loaded into each SDS-PAGE gel. Proteins were transferred to polyvinylidene difluoride (PVDF) membranes (Bio-Rad) with the Trans-Blot Turbo transfer apparatus (Bio-Rad). Membranes were blocked with 5% bovine serum albumin in TBS-T (Tris-buffered saline, 0.1% [vol/vol] Tween) before probing overnight at +4°C with antibodies to the following targets: β-actin (rabbit, horseradish peroxidase [HRP] conjugated, catalog number 5125; Cell Signaling), ATF6 (mouse, catalog number ab122897; Abcam), BiP (rabbit, catalog number 3177; Cell Signaling), CHOP (mouse, catalog number 2895; Cell Signaling), eIF2α (rabbit, catalog number 5324; Cell Signaling), influenza A virus (goat, catalog number ab20841; Abcam), influenza virus NA (rabbit, catalog number GT288; GeneTex), IRE1 (rabbit, catalog number 3294; Cell Signaling), influenza A virus PA (rabbit, catalog number GTX125932; GeneTex), PARP (rabbit, catalog number 9542; Cell Signaling), PERK (rabbit, catalog number 5683; Cell Signaling), phospho-S51-eIF2α (rabbit, catalog number 3398; Cell Signaling), and XBP1s (mouse, catalog number 12782; Cell Signaling). Membranes were washed with TBS-T and incubated with HRP-linked secondary antibodies prior to detection with the Clarity-ECL chemiluminescence reagent (Bio-Rad). All blots were imaged on a Bio-Rad ChemiDoc-Touch system. Molecular weights were determined using protein standards (catalog number P7719; New England BioLabs). Unless indicated otherwise, all Western blot experiments were done twice as independent biological replicates performed on different days.

### Immunofluorescence microscopy.

For immunofluorescence microscopy, A549 cells were seeded on glass coverslips and cultured overnight prior to IAV-CA/07 infection at an MOI of 1 or mock infection. At the indicated times postinfection, cells were fixed with 4% paraformaldehyde and permeabilized with cold methanol as described previously (50). Cells were stained with goat polyclonal antibody to influenza A virus (catalog number ab20841; Abcam Inc., Toronto, ON, Canada), mouse anti-G3BP1 (catalog number 611126; BD Biosciences), mouse anti-PABP (catalog number 10E10; Santa Cruz), rabbit anti-TIAR (catalog number 8509; Santa Cruz), and anti-eIF3A (catalog number 3411; Cell Signaling), followed by Alexa-coupled donkey secondary antibodies (Thermo Fisher Scientific) at a 1:1,000 dilution, along with 5 ng/ml Hoechst dye (blue). Images were captured using a Zeiss AxioImager Z2 microscope. Bars represent 20 μm.

### Semiquantitative *XBP1* mRNA splicing assay.

The semiquantitative *XBP1* mRNA splicing assay was performed as described previously (51). Specifically, total RNA was isolated from treated A549 cells with the RNeasy kit (Qiagen). Five hundred nanograms of total RNA was reversed transcribed using the Maxima H RT kit (Thermo). A 473-bp PCR product spanning exon/intron boundaries was generated using the *XBP1* forward primer 5′-AAACAGAGTAGCAGCTCAGACTGC-3′ and the *XBP1* reverse primer 5′-TCCTTCTGGGTAGACCTCTGGGA-3′. The PCR product was digested overnight with PstI-HF to cleave the unspliced *XBP1* product into XBP1u1 and XBP1u2. The digested PCR product was resolved on a 2.5% agarose gel made with 1× Tris-acetate-EDTA and stained with ethidium bromide (Sigma-Aldrich). The gel was imaged on a ChemiDoc imaging station (Bio-Rad).

### Puromycylation assay.

The puromycylation assay was performed as described previously (31). Cells were treated with 10 μg/ml puromycin for 10 min before lysis. Puromycin is a structural analog of an aminoacyl tRNA, allowing its incorporation into a polypeptide during translation elongation. Lysates were first run on a stain-free gel (Bio-Rad) to image the total protein content. Proteins were then transferred to a PVDF membrane and probed using a mouse monoclonal antibody against puromycin (catalog number MABE343; MilliporeSigma, Etobicoke, ON, Canada).

### Flow cytometry.

A549 cells were mock infected for 1 h at 37°C and washed once with PBS, followed by the addition of fresh IAV infection medium without TCPK-treated trypsin with the indicated treatments. Twenty-three hours after drug treatment, the cells were detached using 10 mM EDTA in PBS, washed with PBS, and stained for 30 min at 4°C with allophycocyanin (APC)-conjugated anti-human HLA-A/B/C (IgG2aκ, catalog number 311410; BioLegend) or an APC-conjugated mouse IgG2aκ isotype control (catalog number 400222; BioLegend). After staining, the cells were washed twice with PBS, fixed with 4% paraformaldehyde in PBS for 30 min at room temperature, washed twice with PBS, and resuspended in 1% BSA–1 mM EDTA–0.05% NaN_3_ in PBS. A549 cells were analyzed using a FACSCanto II instrument (BD) using the same acquisition voltages for all replicates, with at least 23,000 cells acquired per sample. Data analysis was performed using FlowJo v.10.

### CRISPR KO production.

The lentiCRISPR-v2 plasmid was a gift from Feng Zhang (Addgene plasmid 52961 [http://n2t.net/addgene:52961]; RRID Addgene_52961) (52). The lentiCRISPR-v2 plasmids encoding guide RNAs (gRNAs) targeting human PERK or a nontargeting (NT) guide RNA control were cloned with primer sequences designed using the Broad Institute GPP Web portal (https://portals.broadinstitute.org/gpp/public/analysis-tools/sgrna-design). Guide RNA insert sequences are as follows: 5′-GA ATA TAC CGA AGT TCA AAG (PERK guide 1), 5′-GG ACC AAG ACC GTG AAA GCA (PERK guide-2), and 5′-GC ACT ACC AGA GCT AAC TCA (NT guide) (sequence similar to that of the scrambled guide RNA in the pCas-Guide-CRISPRi-Scramble vector from OriGene Technologies). A549 cells were transduced with lentiviruses generated with these vectors at an MOI of 1.0, and stably transduced cells were selected with 1 μg/ml puromycin for 48 h. Resistant cells were seeded onto 96-well dishes for single-cell clone isolation. Knockout clones were confirmed using Western blotting and subsequently used in experiments.

### Viral gene expression and genome replication.

RNA was extracted from infected cells using the RNeasy Plus minikit (Qiagen Inc., Toronto, ON, Canada), and cDNA was generated using Maxima H Minus reverse transcriptase (Thermo Fisher Scientific, Grand Island, NY, USA) in separate reaction mixtures containing the gene-specific primer for 18S rRNA (5′-AGGGCCTCACTAAACCATCC-3′) and either the influenza A virus-specific universal primer Uni12 (5′-AGCAAAAGCAGG-3′) (for viral RNA [vRNA]) or the oligo(dT)_18_ primer (for mRNA). Segment-specific primers were used to analyze IAV genomic RNA and mRNA. Quantitative PCR analysis was performed using PerfeCta SYBR green fast mix (Quanta Bio). Relative initial template quantities were determined using the ΔΔ*C_T_* method. Amplifications were performed using a Bio-Rad CFX Connect instrument and analyzed using Bio-Rad CFX Manager 3.1 software. Primer sequences are presented in Table 1.

**TABLE 1:** Primer sequences for RT-qPCR analysis^*a*^

RT-qPCR target and primer direction	Primer sequence (5′–3′)
5S rRNA
F	GCCCGATCTCGTCTGATCT
R	AGCCTACAGCACCCGGTAT

BiP
F	GCCTGTATTTCTAGACCTGCC
R	TTCATCTTGCCAGCCAGTTG

CHOP
F	ATGAACGGCTCAAGCAGGA
R	GGGAAAGGTGGGTAGTGTGG

EDEM1
F	TTGACAAAGATTCCACCGTCC
R	TGTGAGCAGAAAGGAGGCTTC

ERdj4
F	CGCCAAATCAAGAAGGCCT
R	CAGCATCCGGGCTCTTATTTT

GAPDH
F	ACGAATTTGGCTACAGCAACAGGG
R	TCTACATGGCAACTGTGAGGAGG

HERPUD1
F	AACGGCATGTTTTGCATCTG
R	GGGGAAGAAAGGTTCCGAAG

PR8 HA
F	CTGGACCTTGCTAAAACCCG
R	TCTGGAAAGGGAGACTGCTG

PR8 NA
F	TCACTTGGAATGCAGGACCT
R	CGATTGTTAGCCAGCCCATG

a F, forward; R, reverse; GAPDH, glyceraldehyde-3-phosphate dehydrogenase.

### Statistical analysis.

Statistical analysis was performed on values obtained from at least 3 independent biological replicates with GraphPad Prism 8, using one-way analysis of variance (ANOVA) followed by a Tukey multiple-comparison test or two-way ANOVA followed by Dunnett’s multiple-comparison test. Significance is indicated in the figures (*, *P* value of <0.05; **, *P* value of <0.01; ***, *P* value of <0.001; ****, *P* value of <0.0001).
